# Early life-history predator-prey reversal in two cyprinid fishes

**DOI:** 10.1038/s41598-017-07339-w

**Published:** 2017-07-31

**Authors:** Marek Šmejkal, Roman Baran, Petr Blabolil, Lukáš Vejřík, Marie Prchalová, Daniel Bartoň, Tomáš Mrkvička, Jan Kubečka

**Affiliations:** 1 Institute of Hydrobiology, Biology Centre of the Czech Academy of Sciences, Ceske Budejovice, Czech Republic; 2 Faculty of Science, University of South Bohemia, Ceske Budejovice, Czech Republic; 3 Faculty of Economics, University of South Bohemia, Ceske Budejovice, Czech Republic

## Abstract

Predator-prey relationships are often perceived simply as a situation in which a predator enhances its own fitness while reducing the fitness of its prey. However, this relationship may become reversed when the prey feeds on the juvenile predator stages. We investigated this phenomenon in a model asp (*Leuciscus aspius*; predator)-bleak (*Alburnus alburnus*; prey) relationship. The adhesive asp eggs are available for bleak predation after a spawning event for only tens of seconds before they adhere to the stones, where bleak do not forage. Gut content analysis demonstrated that eggs were utilized in high quantities, especially in the spawning peak of the asp reproductive season. Furthermore, using underwater video, we recorded the bleak feeding efficiency on naturally drifting asp eggs as the percentage of eggs eaten. Within the 40 cm egg trajectory captured by our cameras, total egg mortality was 21.2 ± 2.2% on average. The highest survival chances occurred among eggs drifting in aggregations, since the short drifting distance together with their aggregated distribution satiated bleak and part of the egg aggregation could attach to the spawning ground. This study emphasizes the potential efficiency of predator egg utilization by prey, which may have further consequences in predator-prey dynamics.

## Introduction

Ectotherm vertebrate predators inhabiting aquatic environments commonly utilize the food web over a wider range throughout their lifetime compared to endotherms, since their initial size is usually much smaller and their growth is indeterminate^1–3^. Their vertebrate prey is frequently omnivorous, foraging on small organisms^4^. If the prey of such species is able to utilize predators’ early life stages, such as eggs and larvae, the predator-prey relationship may become reversed, and predators may be foraged upon by large prey individuals^5^. Since prey is naturally numerous, a considerable proportion may be consumed, especially if a large part of the prey population forages on the early life stages of predators^5–7^. Considering the small-sized eggs and larvae available in enormous quantities in many fish groups, some prey individuals may consume more predators in their lifetime than an adult predator consumes its prey^8–10^.

The occurrence of predator eggs and larvae is commonly restricted to a short post-spawning period, which is only when such a reversed situation may occur. However, the easy accessibility of this energetically rich food source may lead to a complete switch from the common diet of the prey to predator eggs and offspring, especially when reproduction occurs early in the season when other food sources are scarce^5, 11, 12^.

In comparison with the long-term dependence of predators on prey availability in predator-prey systems, the short-term predator egg or larvae consumption by prey species likely does not represent a very important food source for the prey. Hence, its absence or low availability would not cause bottom-up limitations in prey populations^13^. This means that the size of the prey population is independent of the egg food source; however, prey may utilize it to a large extent^14, 15^. This can escalate into a positive loop in which high prey abundance may exert high consumption pressure on the early life stages of a predator, which consequently lowers predator recruitment. Hence, numerous prey foraging on their predator may eventually cause considerable changes in the predator-prey dynamics, keeping the predator population low in abundance or triggering more pronounced fluctuations of predators in time^5, 16, 17^.

Contradicting the simple definitions of predator and prey roles in ecosystems, predator-prey reversal was discovered among many species in recent decades^14, 18, 19^. In marine benthic communities, rock lobsters, *Jasus lalandii*, usually represent a keystone predator in diverse ecosystems. However, if its common prey, the whelk (*Burnupena* sp.), greatly outnumbers the rock lobster, it may result in an alternative stable ecosystem where rock lobsters are killed by whelks, which dominate in the alternated ecosystem^14^. Wizen & Gasith experimentally demonstrated how larvae of the ground beetle *Epomis* sp. lure and kill their anuran predator, which otherwise feeds on many insect species^20^. It is likely that predator-prey reversal is most common in aquatic environments, where tiny eggs of many predator fishes are vulnerable to predation by other fish species and invertebrates^21–24^. This phenomenon is potentially very common among fishes and has possible repercussions in commercially harvested waters as well as in protected species ecology.

To investigate predator-prey role reversal, we used a simple model system of two fish species, the asp (*Leuciscus aspius*; predator) and bleak (*Alburnus alburnus*; prey), which are common cyprinid fishes inhabiting inland waters of Central and Eastern Europe^25, 26^. The asp is a long-lived, large, iteroparous predator, migrating to fluvial spawning grounds in the early spring, where it spawns above the open substrate and provides no parental care to its eggs and larvae^26, 27^. The negatively buoyant adhesive eggs are approximately 2 mm in diameter; they are spawned near the water surface and carried by the water current until they adhere to cobbles and stones on the river bottom^27^. Asps do not forage on prey fishes during the spawning season, and first attacks are observed shortly after the termination of spawning^28^. The bleak is a small, short-lived species with a superior mouth position, feeding preferably on emerging insects and zooplankton (in standing water) near the water surface^25, 29, 30^. Adult bleak are oftentimes foraged upon by asps, being their most common prey item^25, 31, 32^.

In the present study, we investigate predator-prey role reversal in the asp-bleak relationship. Specifically, the goals of this study are to (I) analyse whether and to what extent predator-prey role reversal occurs in the asp-bleak relationship by stomach content analysis, (II) analyse whether egg consumption is dependent on bleak size, (III) establish the relative effectiveness of egg consumption using underwater cameras and (IV) model the relationship between egg survival probability and the batch size of drifting eggs using underwater camera data.

## Results

**Figure 1:**
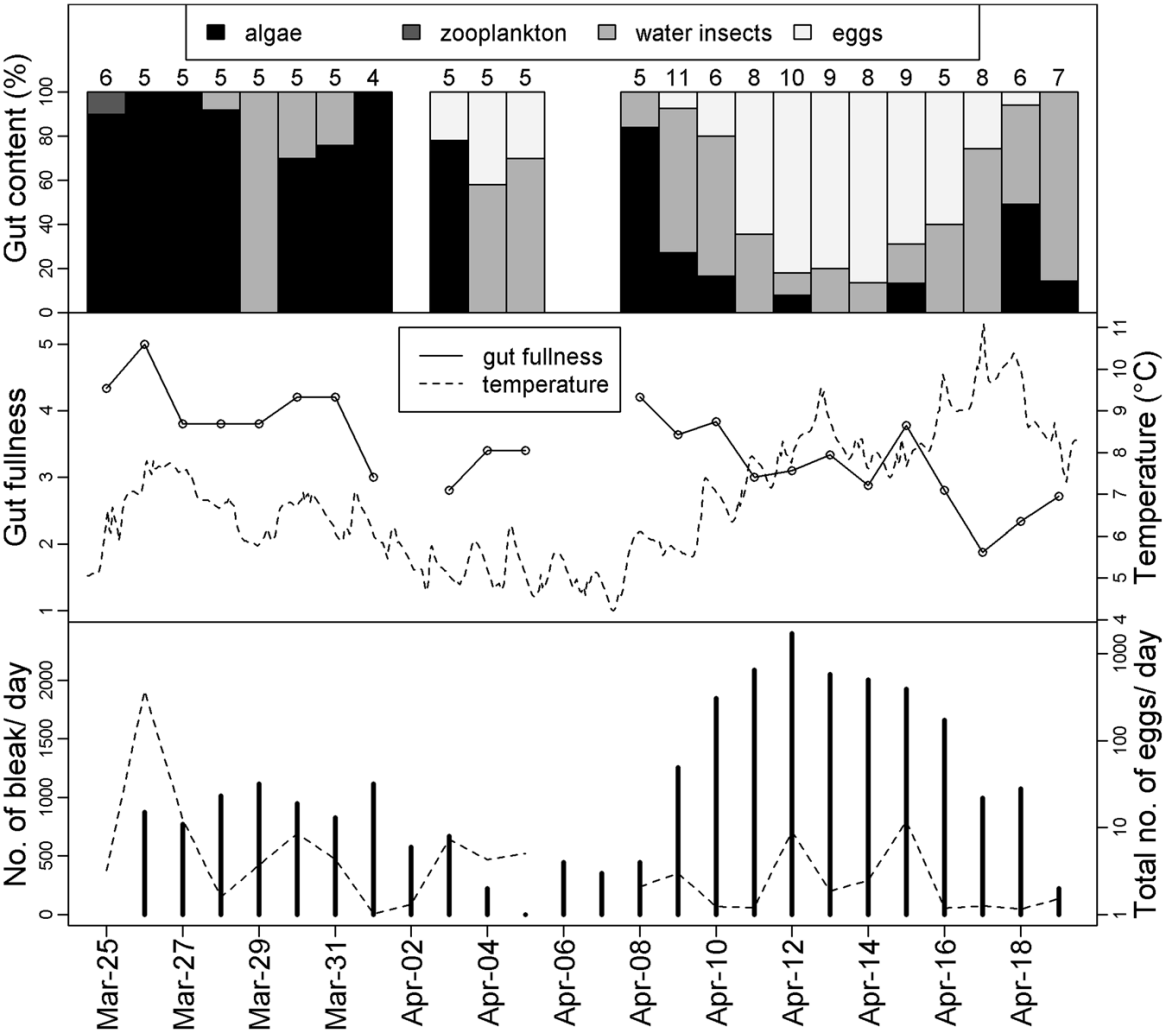
The gut content analysis of bleak (upper and middle panel) and electrofishing estimates of number of bleak within sampling transect (lower panel) in 2015. The numbers above the upper histogram represent the sample size for gut content analysis on a given day. Gut fullness is displayed in the middle panel on a scale of 0–5, with a value of 0 representing empty and a value of 5 fully distended. The temperature is shown by the dashed line. Bottom panel displays the total number of eggs (collected from 12 monitoring tiles, 40 × 40 cm, log 10) and number of bleak in the monitored transect (dashed line).

Gut content analysis demonstrated that asp eggs were an important food source for bleak. During the sampled period, 64 out of the 147 caught bleak individuals contained eggs, with a 31 ± 42% (mean ± SD) contribution to the total amount of food. Other important food sources in this period were water insects (65 individuals, 34 ± 44%), filamentous algae (54 individuals, 34 ± 46%) and zooplankton (two individuals, 1 ± 3%). The proportion of eaten eggs was independent of bleak size (GLM F = 1.29; df = 1; p = 0.257), and eggs prevailed, especially when their abundance in the environment was high (GLM F = 8.54; df = 22; p < 0.001 Fig. 1). Gut fullness was independent of bleak size (GLM F = 0.23; df = 1; p = 0.635), with a significant effect of the date (GLM F = 1.74; df = 22; p = 0.031; Fig. 1).

**Figure 2:**
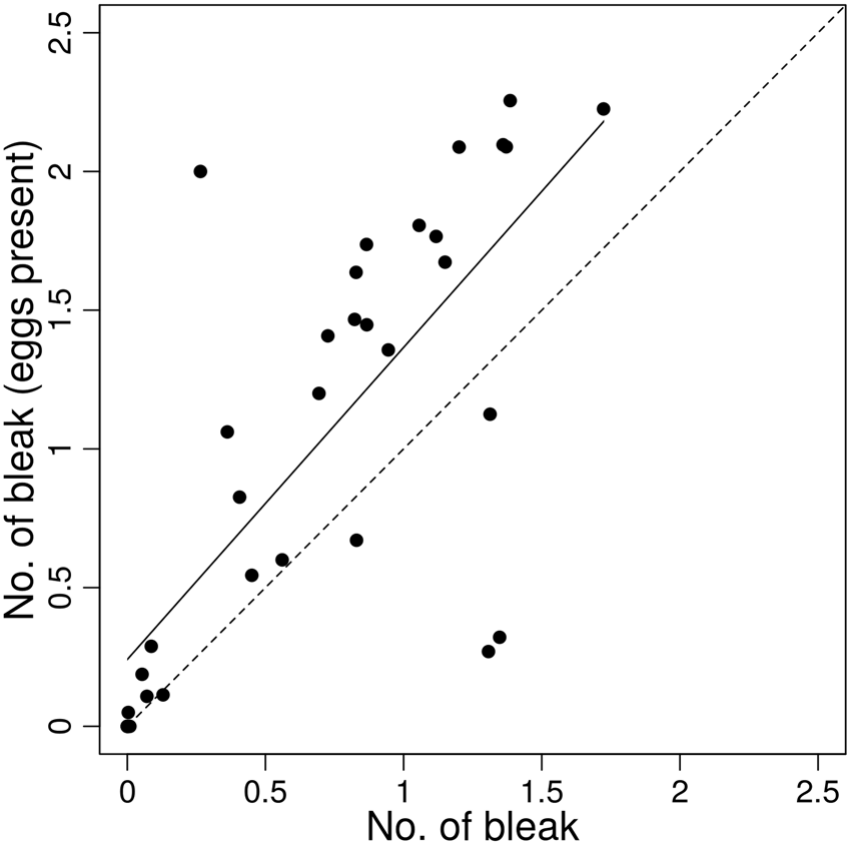
The relationship between the mean number of bleak in camera view during a single video recording and the mean number of bleak in camera view during the same recording when eggs were present in camera view. Dashed line indicates 1:1 ratio, solid line represents trend line and dotted lines represent 95% confidence interval.

One-tailed paired t-test between the average number of bleak and the average number of bleak when at least one egg was drifting in the camera view indicates that bleak were more numerous in the camera view when eggs were drifting (mean ± SD: 0.69 ± 0.53 vs. 1.01 ± 0.80; t (33) = 3.55, p < 0.001; Fig. 2). According to the binomial model, total egg mortality was 21.2 ± 2.2% and bleak foraging efficiency was 53.6 ± 3.2% - estimated from recordings when at least one bleak was present in the camera view (within the 0.42 m detection distance of our cameras).

**Figure 3:**
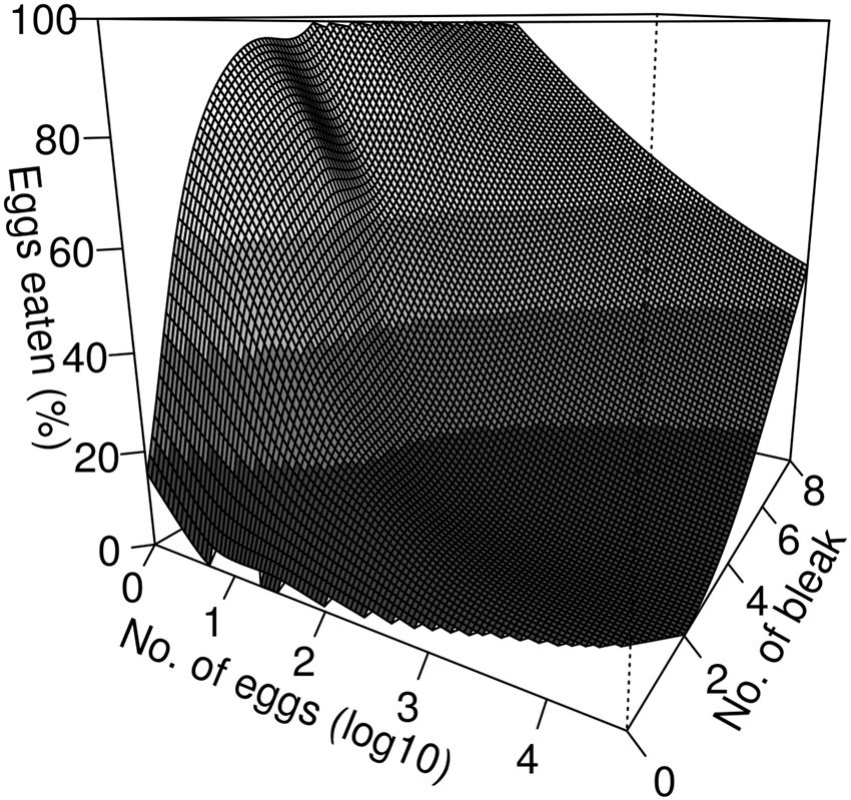
The 3D visualization of the relationship among number of eggs (log 10), number of bleak and percentage of eaten eggs. Shades of grey indicate modelled percentage of eaten eggs in the given scenario (the lowest number of eaten eggs – dark grey, the highest – light grey).

Our logit model, which considers only recordings when eggs were drifting and at least one bleak was within view of the camera, reveals a high probability of eggs being consumed by bleak. The effect of the number of bleak in the model is significant (Wald test χ^2^ (N = 1509) = 73.6; p < 0.001). An increasing number of drifting eggs significantly decreased the probability of being eaten for an individual egg, implying an advantage of aggregation (Wald test χ^2^ (N = 1509) = 102.3; p < 0.001; Table 1; Fig. 3).

**Table 1:** Examples of survival probability of individual eggs computed from the logit model for different numbers of bleak ranging from one to five combined with one, two, three and 10 drifting eggs.

		No. of bleak				
		1	2	3	4	5
No. of eggs	1	0.574	0.469	0.367	0.275	0.199
	2	0.576	0.471	0.368	0.276	0.200
	3	0.577	0.472	0.369	0.277	0.201
	10	0.587	0.482	0.379	0.286	0.208

## Discussion

In this study, we demonstrated that, due to high foraging efficiency, the reversed predator-prey relationship in the studied case may possibly lead to a considerable reduction in the number of eggs surviving until attachment to the substrate (i.e., first tens of seconds in the stream). The gut content analysis revealed that eggs represented a considerable part of the bleak diet in the studied period, especially during the highest spawning activity of asps. From our video records, the bleak egg-foraging efficiency was very high when eggs passed through the camera view, suggesting considerable predation pressure on the predator’s eggs by its prey. Eggs drifting in dense aggregations had a higher probability of survival than those drifting alone or only in small aggregations.

While fish predation on fish eggs has been previously described in many cases (e.g. refs 17, 21, 33 and 34), this seems to be, to our knowledge, the only study in which adhesive eggs are utilized immediately after their release by female fish, before their attachment to the substrate. Commonly studied systems are pelagic or benthic environments, where eggs may be utilized by an egg-eating organism throughout the egg development period^22, 34–36^, and hence the foraging fish may consume an enormous part of the egg production^15, 37^. Despite the short time available, bleak have been shown to utilize the eggs of its predator to a considerable extent. In 2016, the monitoring site was shifted lower due to the presence of air bubbles near the weir, and hence some of the eggs spawned below the weir could have adhered to the substrate before reaching the monitoring site. Hence, we cannot accurately estimate the total predation pressure on asp eggs by bleak, as it is very likely that their survival in the drifting stage may be higher in the uppermost part of the spawning ground, where the drifting aggregations may be denser. We assume that adhered eggs are probably safe from the predation of bleak, which are adapted to foraging near the water surface and do not utilize benthic food sources^25, 29, 30^. We never observed sand or gravel in the bleak gut content analysis, which would otherwise indicate utilizing eggs that had adhered to the substrate (eggs were oftentimes covered with sand particles on monitoring tiles).

In our model, we demonstrated the advantage of drifting in dense aggregations, where there is a higher survival probability for individual eggs. Due to the short drifting period of asp eggs (tens of seconds), aggregation may minimize the individual chances of being chosen by bleak^38–40^.

Female asp specific fecundity is somewhere between 27,500 and 58,500 eggs per kg of body weight, and females appear to release large quantities of eggs in each spawning event^41^. Despite the observed advantage of drifting in aggregations, most of the eggs were recorded singly, and if they appeared simultaneously, it was in very low numbers. They were likely spawned further upstream from the camera position and were diluted by the current before reaching our monitoring site. Alternatively, it is possible that some of those eggs were not actually freshly spawned but eggs that had been detached from the substrate by the strong water current. Those eggs may not be able to settle in a suitable fluvial environment due to the deactivation of the sticky glycoprotein layer with time, and hence they might not survive even if not consumed by bleak^42^. However, we could not discern between detached eggs and scattered freshly spawned eggs in our video records. Given the circumstances, the impact on recruitment may not be as severe as it appears from the bleak egg-consumption efficiency, since some of the eggs may be considered lost when detached from their optimal substrate. On the other hand, the total drifting distance before adhesion to the substrate will influence egg survival. Our estimate of egg survival was computed for the short drifting distance in the camera view, while the actual drifting distance may be several metres^28^. Hence, the proportion of eaten eggs could be considerably higher.

Although we have been counting the number of bleak in the monitored transect in 2015, bleak number seemed to not be strongly related to the egg availability inferred from the monitoring tiles (e.g. high egg abundance did not correspond with high bleak abundance). We believe that this discrepancy may be caused by the transect location: to protect the main spawning site, electrofishing was performed in the lower part of the tributary. Hence, the large number of bleak visually observed in the uppermost part could not be reached by electrofishing.

While we assume that the consumption of asp eggs is necessarily influencing recruitment, it may actually not be so straightforward^9^. Once entering the feeding regime, the early life stages may be limited by density-dependent processes, and the bleak-consumed part of the eggs may not influence actual recruitment, since the survivors may have higher food abundance due to decreased competition and hence higher condition, growth rate and survival^43^. Especially if we consider as successful reproduction offspring that actually reach the reproductive age (4–5 years in asps)^28^, there may not be a difference between bleak-limited and bleak-non-limited recruitment. However, a case study of reproductive success in the common carp, *Cyprinus carpio*, in a lake system in the Mississippi river basin demonstrated that small omnivorous fish effectively control carp recruitment by feeding on eggs and larvae. Carp recruitment was high only in lakes where small omnivorous fish were nearly absent due to winterkills by hypoxia^44^. Similar to our study, a large number of eggs was found in stomachs of egg-feeding species, and eggs disappeared from the spawning ground due to excessive egg-feeding by omnivores^37^.

Although the observed predator-prey reversal in the asp-bleak relationship was demonstrated in only one ecosystem, it is likely a very common phenomenon due to the reproductive migration of both species. Asp migration to the fluvial spawning ground is shortly followed by bleak migration into similar spawning grounds, where it spawns few weeks after the asp^45^. Hence, the first bleak migrants into the stream encounter an abundance of fat-rich food represented by asp eggs^46^.

To conclude, we demonstrated in this study a very high prey foraging efficiency on predator eggs, which may have further consequences on the predator-prey dynamics. This study seems to be the first describing the impacts of egg-eating predators on the eggs of lithophilic spawners before adhesion to the substrate. Further, we provide a basic model showing that in this predator-prey reversed relationship; egg aggregation provides survival benefits for individual eggs. We believe that future research should focus on the assessment of the damage done to asp eggs by bleak and the benefits for prey species. In addition, and most importantly, does the reversed predation influence predator recruitment and to what extent?

## Materials and Methods

### Study site

The study was conducted in the main tributary of the Želivka Reservoir, located at 49°578497′ N, 15°251671′ E, Czech Republic. The reservoir is 39.1 km long with an area of 1602 ha at a maximum water level of 381.7 m above sea level. Approximately 2000 asps migrate from the reservoir to spawn in the early spring (mid-March to mid-April) and release a vast amount of eggs and milt into a 100 m long tributary within a one-month long spawning period^28, 47^. Further upstream, the migration is restricted by a weir, and downstream, spawning is limited by an inadequate water current and the substrate composition; hence, spawning along with egg predation can be monitored within the short study site^27^. Thousands of bleak individuals were repeatedly observed in the tributary at the peak of the spawning period. This seems to be the most abundant egg-eating species in this system^28^. Hence, we focus here on this species, although other potential egg-eating species were also observed (see bleak electrofishing below).

### Bleak electrofishing and gut content analysis

**Figure 4:**
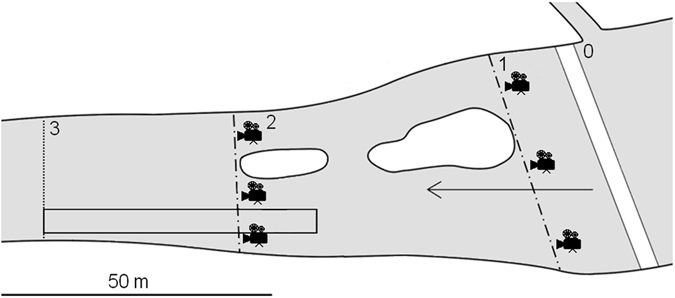
Schematic representation of the spawning ground located downstream of the weir. The space between the weir (0, solid lines) and the dotted line represents the estimated spawning ground^27^. Electrofishing was performed within a 50 m transect visualized by the rectangle. Dot-dashed lines represent position of video monitoring sites for 2015 (site no. 1) and 2016 (site no. 2). Within each site symbolized by camera illustration, three cameras were placed randomly. At sites no. 1, 2 and 3, water velocity was computed using daily mean water discharge data. Four monitoring tiles were deployed at each site, and eggs were counted and removed daily. Arrow shows the direction of flow. The figure was generated by the software ArcMap, version 10.2.2^51^.

Electrofishing was performed from 25 March to 19 April 2015 daily at 3 p.m. by an electrofishing boat (electrofisher EL 65 II GL DC, Hans Grassel, Schönau am Königsee, Germany, 13 kW, 300/600 230 V). Due to rainy weather conditions, sampling was not conducted on three days (2, 6 and 7 April) due to safety reasons. A 50 m long and 6 m wide transect was sampled (Fig. 4). Electrofishing was not performed further upstream in order to not damage the major spawning ground undergoing long-term monitoring.

Due to the fragile nature of bleak and the long-term sampling schedule, we decided to only count bleak to avoid damaging a large number of egg-eating individuals by hand nets (bleak scales are very easy to remove during handling). The number of bleak and other omnivorous fish species was estimated each day within the defined transect. In total, 9,589 bleak, 133 roach (*Rutilus rutilus*) and 76 bream (*Abramis brama*) were recorded. A subsample of bleak within the transect (5–11 individuals) was measured (nearest mm), weighed (nearest 0.1 g) and collected for gut analysis (standard length 108 ± 14 mm, weight 13.9 ± 6.0 g, mean ± standard deviation (SD)) during every electrofishing day (total sample: 147 bleak individuals). Fish were anaesthetised using a lethal dose of MS–222 and immediately processed in the field laboratory. A gut fullness index was assigned to each individual fish on a scale of 0 to 5; 0 indicating empty and 5 fully distended^48, 49^. Gut content was classified into four food categories: asp eggs, filamentous algae, water insects and zooplankton. The relative volume of each food category was estimated visually as a percentage of the total gut content volume of each gut.

### Monitoring of asp eggs

We monitored the egg density on standardized concrete tiles (Fig. 4) to assess the main asp spawning period. A hole was drilled in the centre of each tile, which was then attached to a rope with a float to facilitate monitoring. At three sites on the spawning ground, a total of 12 concrete tiles, 40 × 40 cm, were deployed. The surrounding spawning substrate consisted of large stones and pebbles. The tiles were checked daily at 7 p.m. for new eggs and were cleaned after eggs were counted.

### Egg detection efficiency in video records

In order to reveal whether an observer is able to detect all eggs drifting in the camera view, an experiment was conducted on the studied site from 8 to 9 April 2017. Random number of asp eggs (0–35) was released upstream from the camera position so the eggs drifted in the camera view. Altogether, 100 replicates were performed. Egg detection efficiency was evaluated using double-blind methodology. The observer of the video had no prior knowledge of the number of released eggs and the same person analysed all records obtained in this study. Egg detection efficiency was estimated to be 98.05 ± 4.40% based on a comparison of counted vs. released numbers of asp eggs.

### Video monitoring

Asp spawning activity was visually observed each day and inferred from the number of eggs on the monitoring tiles. Three underwater cameras (Epoque Edivecam, Epoque World Co., Ltd., Japan) were randomly placed around monitoring sites oriented downstream to record bleak foraging on days when bleak presence at the spawning ground was visually observed and asps exhibited high spawning activity (10–12 April 2015 and 5–11 April 2016, Fig. 4). Cameras were mounted on the holder with weights and their detection range covered approximately the upper two-thirds of the water column. The distance at which bleak and eggs could be identified was 42 ± 3 cm (mean ± SD, estimated for each recording day). Recording occurred between 1 and 3 p.m. Altogether, three underwater videos (total time 02:51:45 (hh:mm:ss)) were recorded in 2015, and 31 videos (total time 29:40:45) were recorded in 2016. Our methodology was limited by air bubbles originating from the presence of the weir in 2016, which prevented the use of cameras directly below the weir, and in 2015 due to higher water discharge (Fig. 4).

### Water temperature and discharge

Along with fish monitoring, two data loggers (TidbiT v2, Onset, USA) measuring water temperature were placed in the tributary and recorded the temperature with hourly frequency in 2015 and 2016. To demonstrate slowdown of the water current contributing to the egg adhesion to the substrate, we computed water discharge for three consecutive sites along the spawning ground profile. The water velocity was estimated to be 22 ± 6 cm s^−1^ and 34 ± 9 cm s^−1^ (mean ± SD; 2015 and 2016 seasons, respectively) at the first site, 18 ± 5 cm s^−1^ and 27 ± 7 cm s^−1^ at the second site and 11 ± 3 cm s^−1^ and 16 ± 4 cm s^−1^ at the third monitoring site (Fig. 4). The estimates are based on daily data on total water discharge provided by the river authority (Povodí Vltavy, s.p.).

### Ethics

The field sampling and experimental protocols used in this study were performed in accordance with the guidelines of and with permission from the Experimental Animal Welfare Commission under the Ministry of Agriculture of the Czech Republic (Ref. No. CZ 01679). All methods were approved by the Experimental Animal Welfare Commission under the Ministry of Agriculture of the Czech Republic.

### Video analysis

The video records were used to estimate the capture efficiency of bleak. Bleak were analysed only in records where they were close enough to be discerned from different species inhabiting the studied system. In each video, every recording in which one or more separate eggs (further referred to as aggregation) were drifting was noted, along with the number of bleak within camera view and the number of successfully eaten eggs by bleak. A bleak was counted as present when it was clearly identified and at least its head was visible in the camera view (e.g., more than half of the bleak without its head in view was not counted as present, while only the head being visible was counted as present). The number of bleak in the camera view was counted every 15 seconds to estimate the bleak presence in the given record, and the average was used as a characteristic of each video record. Another average bleak number was calculated for the recordings in which eggs were observed in order to determine whether bleak are attracted by drifting eggs.

### Statistical analysis

Two general linear model (GLM) analyses with the explanatory variables bleak length and sampling date were performed to test (I) the proportion of eggs in the total diet and (II) whether gut fullness was dependent on the explanatory variables used.

To reveal whether there is a relationship between number of bleak and number of drifting eggs (egg-finding behaviour), one-tailed paired t-test was used. For each video record, the average number of bleak in the camera view in moments when eggs were drifting was computed for each video record and was related to the average number of bleak computed from counts spaced at 15 s intervals. Egg-finding behaviour could be identified by the former value being generally higher than the latter.

To study the probability of eggs being eaten, we can assume that the total number of eaten eggs (*X*_*i*_) in one observed video follows the binomial distribution with parameters *n*_*i*_ and *p*, where *n*_*i*_ is the total number of eggs in the *i* ^*th*^ video and *p* is the probability of eggs being eaten. This assumption means that all eggs are taken as independent observations. This assumption is not satisfied because, for instance, the probability of catching an egg when two eggs are observed in the video differs from catching an egg when one egg is observed. Hence, we separately computed the probability (*p*_*k*_) for *k* observed eggs drifting in the video simultaneously. The index *k* is taken from 1 to 10 only to avoid rare situations when the eggs satiated the bleak. The maximum likelihood estimator of *p*_*k*_ follows the binomial model ${\hat{p}}_{k}^{l}={X}_{i}^{k}/{n}_{i}^{k}$, where ${X}_{i}^{k}$ is the number of eaten eggs with *k* observed eggs in the video and ${n}_{i}^{k}$ is the total number of times the *k* eggs are observed. In the binomial model, we can easily pool the observations from different samples together because ${\sum }_{i}{X}_{i}^{k}$ also has a binomial distribution with parameters ${\sum }_{i}{n}_{i}^{k}$ and *p*_*k*_. In addition, we thus obtain a maximum likelihood estimator of the parameter *p*_*k*_ as ${\hat{p}}_{k}={\sum }_{i}{X}_{i}^{k}/{\sum }_{i}{n}_{i}^{k}$_._

To obtain an estimator of *p*, we must use the theorem of complete probability, i.e.,

1 $$\hat{p}={\sum }_{k}{\hat{p}}_{k}\ast {\hat{q}}_{k}$$

where ${\hat{q}}_{k}=\frac{{\sum }_{i}{n}_{i}^{k}}{N}(N={\sum }_{k}{\sum }_{i}{n}_{i}^{k})$ is the estimate of the probability of *k* eggs appearing in the video. This simplifies into

2 $$\hat{p}=\frac{{\sum }_{k}{\sum }_{i}{X}_{i}^{k}}{N}$$

The standard deviation of this estimator can be computed straightforwardly using the fact that ${\sum }_{i}{n}_{i}^{k}$ is distributed according to the multinomial distribution. This can be simplified into ${\sum }_{i}{n}_{i}^{k}={n}^{k}$ and ${\sum }_{i}{X}_{i}^{k}={X}^{k}$. The standard deviation is then computed as

3 $$SD(\hat{p})=\sqrt{{\sum }_{k}(\frac{{X}^{k}{n}^{k}N-{X}^{k}{({n}^{k})}^{2}+{X}^{k}{({n}^{k})}^{2}N-{({X}^{k})}^{2}N+{({X}^{k})}^{2}{n}^{k}+{({X}^{k})}^{2}{n}^{k}N-{({X}^{k}{n}^{k})}^{2}}{{({n}^{k})}^{2}{N}^{3}})}-2\sum {\sum }_{k < l}\frac{{X}^{k}{X}^{l}}{{N}^{3}}$$

The effect of the number of bleak and the number of eggs in a single video observation on the probability of predation on eggs was further evaluated using a classical logit model. Here, the observations with many eggs (more than 10) were also considered, and the dependence on the number of eggs is here taken into consideration in one factor.

Statistical analyses were performed in Statistica software (Statistica, Inc., StatSoft, Tulsa, Oklahoma, USA) and R software version 3.2.3^50^.
